# Characteristics of liver virtual non-contrast images from three-phase dynamic CT obtained with second-generation dual-layer spectral CT

**DOI:** 10.1016/j.ejro.2026.100781

**Published:** 2026-06-19

**Authors:** Yuji Tachibana, Noritaka Kamei, Tomoaki Shiroo, Yoshiki Asayama

**Affiliations:** a Department of Radiological Sciences, Faculty of Medicine, Fukuoka International University of Health and Welfare, 3-6-40 Momochihama, Sawara-ku, Fukuoka City, Fukuoka 814-0001, Japan; b Department of Radiology, Faculty of Medicine, Oita University, 1-1 Idaigaoka, Hasama-machi, Yufu City, Oita 879-5593, Japan; c Radiology Department, Division of Medical Technology, Oita University Hospital, 1-1 Idaigaoka, Hasama-machi, Yufu City, Oita 879-5593, Japan

**Keywords:** Liver, Computed tomography, Dual-Layer Spectral CT, Virtual non-contrast images Image quality

## Abstract

**Objective:**

To clarify the characteristics of virtual non-contrast (VNC) images generated from each phase of triphasic dynamic liver computed tomography (CT) using second-generation dual-layer spectral CT (DLCT).

**Methods:**

A second-generation DLCT system, the Spectral CT 7500, was used for the three-phase dynamic liver CT examination of 31 patients from August 2024 to February 2025. VNC images were generated from the arterial phase (AP), portal venous phase (PVP), and equilibrium phase (EP). For a quantitative evaluation, the agreement between liver parenchymal CT values from the VNC images created from the three phases and the true non-contrast (TNC) images was assessed by a Bland-Altman analysis (pre-set clinical limit: ±10 HU). In the qualitative evaluation, five parameters (vascular remnants, visibility of the portal and hepatic veins, image noise, image distortion, and overall image quality) were assessed using a four-point rating scale.

**Results:**

In the quantitative analysis, the mean difference (bias) between the VNC and TNC images was −2.19 for AP, −0.14 for PVP, and −2.11 HU for EP, and the 95% limits of agreement were within the pre-set clinical limit for all phases. In the qualitative analysis, vascular remnants were significantly more pronounced in the AP and PVP compared to the EP. Consequently, vessel visibility and overall image quality were significantly higher in the VNC images derived from the EP.

**Conclusion:**

Although incomplete removal of vascular contrast enhancement was noted (arterial in the AP and venous in the PVP/EP), VNC images from DLCT are quantitatively interchangeable with TNC images.

## Highlights

• Quantitative VNC attenuation across all phases (AP, PVP, EP) agreed well with TNC within acceptable limits.
• Equilibrium phase VNC scored highest qualitatively due to more accurate iodine removal than AP and PVP.
• VNC holds potential to eliminate TNC scans, but understanding phase-specific characteristics is clinically vital.

## 1 Introduction

Computed tomography (CT) has become widely adopted worldwide due to its high spatial resolution and diagnostic utility, and the number of examinations continues to increase [1], [2]. In the abdominal region, it is used for the detection and qualitative diagnosis of hepatic neoplastic lesions, such as hepatocellular carcinoma and metastatic liver cancer. Furthermore, in these diagnoses, multiphase dynamic CT using contrast agents is indispensable [3], [4], [5], [6].

In recent years, CT technology has advanced significantly. Among these advancements, dual-energy CT (DECT) has garnered attention for its ability to separate materials and perform image analysis by capturing images using two different X-ray energies [7], [8]. One important application of DECT is virtual non-contrast (VNC) imaging. VNC is a technology that virtually removes iodine components from post-contrast imaging data to generate images resembling unenhanced CT scans. If VNC images are comparable to true non-contrast (TNC) images, pre-contrast TNC scans can be omitted. This results in significant clinical benefits, including substantial reductions in patient radiation exposure and shorter examination times [8], [9].

While multiple technologies exist for DECT systems, one approach is dual-layer spectral CT (DLCT). DLCT enables the collection of spectral data from a single radiation source simultaneously, as it can separate the X-ray energy spectrum on the detector side [10]. This technology has continued to evolve, leading to the introduction of second-generation DLCT systems featuring improved detector performance and image reconstruction algorithms, which have enhanced material separation and analysis capabilities [11], [12], [13]. The reliability of VNC quality in DLCT has already been reported in several studies [14], [15], [16]. However, the accuracy of VNC images generated using this latest second-generation DLCT has not been verified, and the characteristics of VNC images derived from each phase of dynamic liver CT remain unclear.

The purpose of this study is to evaluate the quantitative and qualitative value of VNC images derived from a second-generation DLCT of each phase of a three-phase dynamic CT scan of the liver compared to the TNC images.

## 2 Methods

### 2.1 Patients

This was a retrospective study approved by the institutional review board of Oita University Hospital (approval number: 3211; September 1, 2025), and the requirement for informed consent was waived. We included patients who underwent triphasic dynamic liver CT using a second-generation dual-layer spectral CT system (Spectral CT 7500; Philips Healthcare, Amsterdam, Netherlands) at our institution between August 2024 and February 2025. The exclusion criteria were as follows: (1) history of liver surgery (e.g., hepatectomy) or local therapies (e.g., radiofrequency ablation or transarterial chemoembolization); (2) presence of artifacts affecting image evaluation; (3) obvious fatty liver disease, mean hepatic attenuation < 40 HU (Hounsfield unit) on TNC images [17]; and (4) incomplete triphasic imaging protocols.

### 2.2 CT protocol

All patients were scanned using a second-generation dual-layer spectral CT system (Spectral CT 7500). The acquisition parameters were as follows: tube voltage, 120 kVp; rotation time, 0.3 s; collimation, 0.625 × 128 mm; and helical pitch, 1.0. Hybrid iDose 5 (Philips Healthcare) was used for the reconstruction method, and reconstruction was performed at noise level 23. For contrast-enhanced CT, one of the following non-ionic iodinated contrast medium: iopamidol (Iopamiron; Bayer Schering Pharma, Leverkusen, Germany), iohexol (Omnipaque, General Electric Company, Boston, MA, USA), or iomeprol (Iomeron; Bracco, Milan, Italy) was injected intravenously. The contrast dose was adjusted to 600 mgI/kg of body weight and injected over 30 s using a power injector, followed by a saline flush. The scan timing for the arterial phase (AP), portal venous phase (PVP), and equilibrium phase (EP) was determined using a bolus-tracking technique. The AP scan was triggered 20 s after the attenuation of the descending aorta reached 100 HU. The PVP and EP scans were obtained at 70 and 180 s after the injection, respectively.

### 2.3 Quantitative image analysis

For quantitative image quality assessment, we compared the CT attenuation values between TNC images and VNC images derived from the AP, PVP, and EP: VNC(AP), VNC(PVP), and VNC(EP). A radiological technologist with 16 years of experience performed the quantitative analysis. VNC images were generated from each phase using the specific workstation provided with the scanner (Philips Healthcare). First, the CT attenuation (in HU) of the liver parenchyma was measured on TNC and all VNC images. Regions of interest (ROIs) were placed on the liver parenchyma, avoiding major vessels and large branches to minimize the influence of blood flow. Specifically, three ROIs (size, 150 mm²) were placed, and the mean attenuation was calculated. The ROI positions were kept identical across all phases and TNC images. All measurements were performed using images reconstructed with a slice thickness and interval of 1 mm.

### 2.4 Qualitative image analysis

For qualitative image quality evaluation, two board-certified radiologists (with 23 and 30 years of experience, respectively) independently evaluated the VNC(AP), VNC(PVP), and VNC(EP) images. The evaluation criteria included vascular remnants, visibility of the portal and hepatic veins, image noise, image distortion, and overall image quality. The CT datasets were randomized, and the readers were blinded to the imaging phases. The analysis was performed using a four-point subjective scale.

Vascular remnants were graded as follows: 1, marked remnants (vessels not completely removed); 2, moderate remnants; 3, minimal remnants; and 4, no remnants observed. The visibility of the portal and hepatic veins was assessed as follows: 1, main branches (right and left) not visible; 2, main branches visible; 3, segmental branches visible; and 4, subsegmental branches visible. Image noise, image distortion, and overall image quality were evaluated on a four-point scale: 1, unacceptable; 2, acceptable; 3, good; and 4, excellent. Images reconstructed with a slice thickness and interval of 1 mm were used for evaluation.

### 2.5 Statistical analysis

All quantitative values are expressed as means ± standard deviations. For quantitative evaluation, Bland-Altman analysis was performed to assess the agreement between the CT attenuation of the liver parenchyma in VNC(AP), VNC(PVP), and VNC(EP) images and TNC images. The mean difference (bias) between the two measurements (VNC minus TNC) was defined as the systematic error, and the standard deviation (SD) of the difference was used as an indicator of variability. The CT values of the erector spinae were used as SD. The 95% limits of agreement (LoA) were calculated as bias ± 1.96 × SD. The clinically acceptable range was set at ±10 HU based on prior studies [14], [15].

For qualitative evaluation, the scores of the VNC images were compared using the Friedman test, followed by the Wilcoxon signed-rank test for post-hoc comparisons. Inter-observer agreement was evaluated using Cohen’s quadratic weighted kappa statistics. The degree of agreement was interpreted based on k values as follows: ≤ 0.20, poor; 0.21–0.40, fair; 0.41–0.60, moderate; 0.61–0.80, good; and ≥ 0.81, excellent [18]. A p-value < 0.05 was considered statistically significant. For multiple comparisons, the Bonferroni correction was applied.

All statistical analyses were performed with EZR (Saitama Medical Center, Jichi Medical University, Saitama, Japan), which is a graphical user interface for R (The R Foundation for Statistical Computing, Vienna, Austria). More precisely, it is a modified version of R Commander designed to add functions frequently used in biostatistics [19].

## 3 Results

**Fig. 1:**
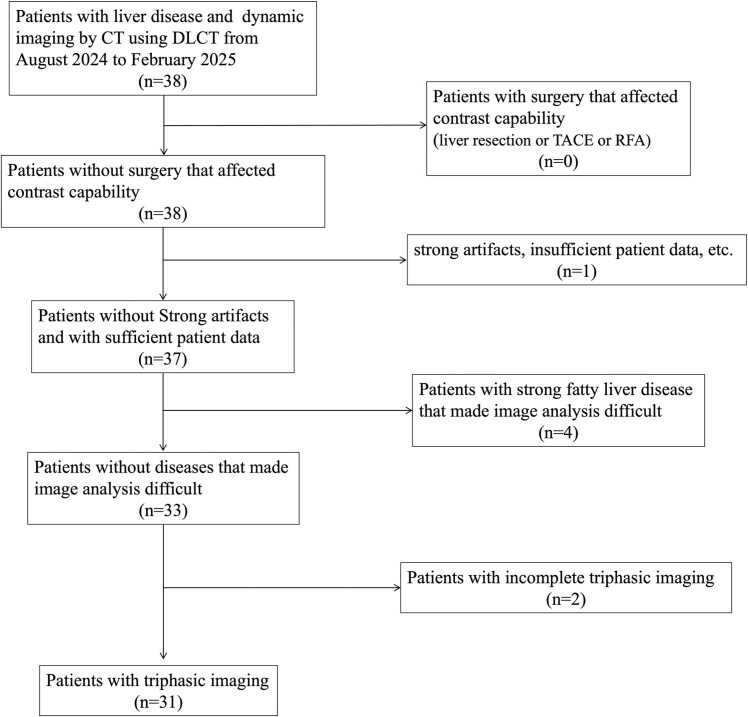
The patient selection flow chart. CT: computed tomography; DLCT: dual-layer computed tomography; TACE: transcatheter arterial chemoembolization; RFA: radiofrequency ablation.

Fig. 1 shows the flowchart of patient selection. A total of 38 patients with liver disease who underwent dynamic liver CT using DLCT were initially identified. None of the patients had a history of liver surgery or local therapy. Of these, seven patients were excluded based on the following criteria: one with severe artifacts, four with fatty liver, and two with incomplete triphasic imaging (dual-phase only). Consequently, 31 patients were included in the final analysis. The patient characteristics are summarized in Table 1.

**Table 1:** The patients’ baseline characteristics.

Characteristic	
Sex (male: female)	16:15
Age (y)	68.84 ± 15.34
Body height (cm)	160.72 ± 7.92
Body weight (kg)	60.20 ± 12.84
Body mass index (kg/m^2^)	23.25 ± 4.20

Note - Data are the mean ± standard deviation

### 3.1 Quantitative image analysis

**Fig. 2:**
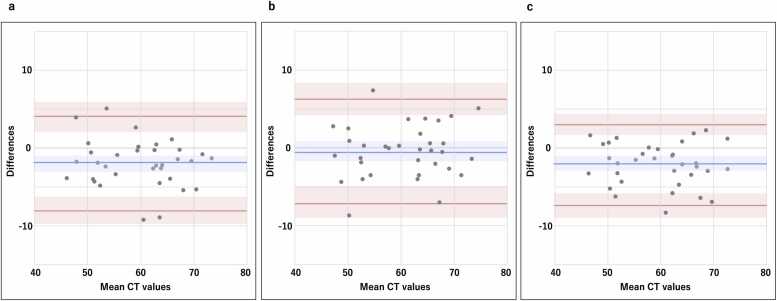
Agreement analysis of liver parenchyma CT Values: VNC vs TNC. a: VNC(AP) vs TNCb: VNC(PVP) vs TNCc: VNC(EP) vs TNC CT: computed tomography; VNC(AP): virtual non contrast for arterial phase, VNC(PVP): virtual non contrast for portal vein phase, VNC(EP): virtual non contrast for equilibrium phase.

**Fig. 3:**
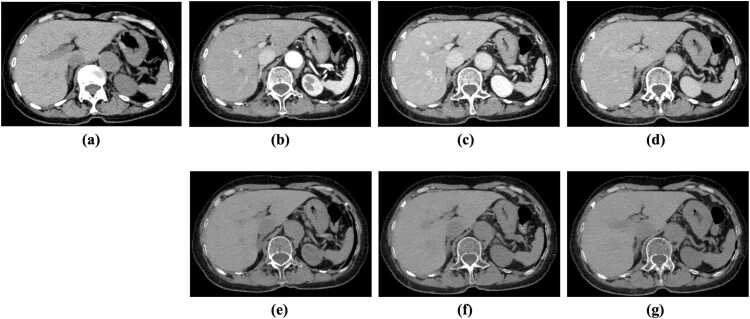
One of the clinical cases with TNC image and VNC images for quantitative evaluation created from the three contrast-enhanced phases. A woman who was 72 years when image. (a) TNC, (b) AP, (c) PVP, (d) EP, (e) VNC(AP), (f) VNC(PVP), (g) VNC(EP). The CT value of the liver parenchyma on the TNC image was 70.7. The CT values on the VNC images were 65.3 on VNC(AP), 63.7 on VNC(PVP), and 64.3 on VNC(EP).

Table 2 summarizes the image analysis results and radiation dose indices for the TNC and triphasic contrast-enhanced acquisitions. Table 3 presents the CT attenuation values of the liver parenchyma on TNC images and the VNC(AP), VNC(PVP), and VNC(EP) images. The results of the Bland-Altman analysis assessing the agreement between TNC and VNC images are shown in Table 4 and Fig. 2. The mean difference (bias) between the VNC and TNC images was −2.19 for VNC(AP), −0.14 for VNC(PVP), and −2.11 HU for VNC(EP), and the 95% LoA were −8.04–3.65 for VNC(AP), −6.22–5.95 for VNC(PVP), and −7.39–3.17 for VNC(EP). Although a negative bias (underestimation) was observed in all three VNC phases, the 95% LoA remained within the predefined clinically acceptable range (±10 HU). Fig. 3 shows representative examples of a clinical TNC image and VNC images for quantitative evaluation created from the three contrast-enhanced phases.

**Table 2:** the image analysis results and radiation dose indices for the TNC and triphasic contrast-enhanced acquisitions.

	TNC	AP	PVP	EP
CT attenuation (HU)				
Abdominal aorta	43.70 ± 5.63	406.52 ± 73.56	180.04 ± 21.20	129.52 ± 18.48
Common hepatic artery	39.10 ± 5.77	374.73 ± 67.36	162.34 ± 21.91	117.94 ± 24.34
Main portal vein	40.21 ± 5.73	164.97 ± 47.73	190.99 ± 25.55	130.62 ± 19.26
Inferior vena cava	44.67 ± 11.18	140.74 ± 32.85	157.10 ± 28.63	125.82 ± 24.09
Liver parenchyma	61.47 ± 7.61	84.78 ± 12.77	125.27 ± 12.94	98.86 ± 8.12
Image noise (HU)	12.21 ± 2.40	12.49 ± 2.56	12.81 ± 2.76	12.10 ± 3.32
CTDI_vol_ (mGy)	17.66 ± 7.52	16.68 ± 5.61	16.85 ± 5.75	16.95 ± 5.46
SSDE (mGy)	25.71 ± 10.22	24.18 ± 6.71	24.45 ± 6.70	24.57 ± 6.39

Note - Data are the mean ± standard deviation. CT: computed tomography, HU: Hounsfield unit, CTDI_vol_: volume computed tomography dose index, SSDE: size-specific dose estimates, TNC: true non contrast, AP: arterial phase, PVP: portal vein phase, EP: equilibrium phase.

**Table 3:** CT attenuation values of the liver parenchyma on TNC and VNC images.

	TNC	VNC (AP)	VNC (PVP)	VNC (EP)
CT attenuation (HU)				
Abdominal aorta	43.70 ± 5.63	52.02 ± 6.50	42.85 ± 4.19	43.84 ± 6.95
Common hepatic artery	39.10 ± 5.77	51.41 ± 10.44	44.52 ± 8.61	40.02 ± 8.47
Main portal vein	40.21 ± 5.73	43.90 ± 7.58	44.13 ± 6.15	41.84 ± 4.26
Inferior vena cava	44.67 ± 11.18	42.08 ± 4.69	42.21 ± 4.90	40.95 ± 8.70
Liver parenchyma	61.47 ± 7.61	59.34 ± 7.41	60.78 ± 6.89	59.12 ± 7.11
Image noise	12.21 ± 2.40	9.64 ± 2.00	9.64 ± 2.04	9.38 ± 2.71

Note - Data are the mean ± standard deviation. CT: computed tomography, HU: hounsfield unit, TNC: true non contrast, VNC(AP): virtual non contrast for arterial phase, VNC(PVP): virtual non contrast for portal vein phase, VNC(EP): virtual non contrast for equilibrium phase.

**Table 4:** Bland-Altman analysis assessing the agreement between TNC and VNC images.

	TNC vs VNC (AP)	TNC vs VNC (PVP)	TNC vs VNC (EP)
Bias	−2.19 ± 2.98	−0.14 ± 3.10	−2.11 ± 2.69
ULoA	3.65	5.95	3.17
LLoA	−8.04	−6.22	−7.39

Note - Data are the mean ± standard deviation. ULoA: upper limit of agreement, LLoA: lower limit of agreement, VNC(AP): virtual non contrast for arterial phase, VNC(PVP): virtual non contrast for portal vein phase, VNC(EP): virtual non contrast for equilibrium phase, TNC: true non contrast.

### 3.2 Qualitative image analysis

**Fig. 4:**
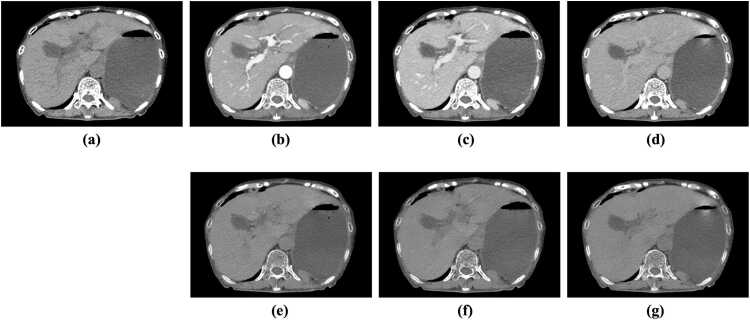
One of the clinical cases with TNC image and VNC images for qualitative evaluation created from the three contrast-enhanced phases. A woman who was 80 years when image. (a) TNC, (b) AP, (c) PVP, (d) EP, (e) VNC(AP), (f) VNC(PVP), (g) VNC(EP). In the evaluation of vascular remnants, VNC(AP) images scored 2.5 points, VNC(PVP) images scored 3 points, and VNC(EP) images scored 4 points. VNC(AP) and VNC(PVP) images show prominent residual vessels, but no residual vessels are present in VNC(EP) images.

Table 5 summarizes the results of the qualitative image analysis, and Table 6 shows the distribution of subjective scores assigned by the two radiologists. The Friedman test revealed significant differences among the three VNC phases regarding vascular remnants, visibility of the portal and hepatic veins, and overall image quality. In post-hoc pairwise comparisons, significant differences were observed between VNC(AP) and VNC(EP), and between VNC(PVP) and VNC(EP) in terms of vascular remnants and vessel visibility. For overall image quality, a significant difference was found between VNC(PVP) and VNC(EP). No significant differences were observed in the other comparisons. In all evaluations, the kappa coefficients exceeded 0.5, indicating moderate to good inter-observer agreement. Fig. 4 shows representative examples of a clinical TNC image and VNC images for qualitative evaluation created from the three contrast-enhanced phases.

**Table 5:** The comparison between VNC images by qualitative image analysis.

	VNC (AP)	VNC (PVP)	VNC (EP)	*p* value (Friedman test)	*p* value VNC (AP) vs VNC (PVP)	*p* value VNC (AP) vs VNC (EP)	*p* value VNC (PVP) vs VNC (EP)
Vascular Remnants	3.00 [3]	3.00 [3]	3.50 [3,4]	＜0.01	0.172	＜0.01	＜0.01
Visibility of the portal vein/hepatic vein	2.50 [2,3]	3.00 [2–3.5]	3.00 [2.5–4]	＜0.01	0.294	＜0.01	0.015
Image noise	4.00 [3.25–4]	4.00 [3.5–4]	4.00 [3.5–4]	0.717	0.348	0.778	0.657
Image distortion	4.00 [4]	4.00 [4]	4.00 [4]	0.607	1	1	1
Overall image quality	3.00 [3]	3.00 [3]	3.00 [3–3.75]	＜0.01	0.235	0.080	＜0.01

Note - Data are presented as medians with interquartile ranges in brackets. VNC(AP): virtual non contrast for arterial phase, VNC(PVP): virtual non contrast for portal vein phase, VNC(EP): virtual non contrast for equilibrium phase.

**Table 6:** The distribution of subjective image scoring for different protocols by two radiologists.

Parameter	Reviewer 1	Reviewer 2	*Agreement (κ)
Vascular Remnants (1/2/3/4)			
VNC (AP)	0/2/29/0	0/3/27/1	0.652
VNC (PVP)	0/7/23/1	0/6/24/1	0.923
VNC (EP)	0/1/15/15	0/1/14/16	0.949
Visibility of the portal vein/hepatic vein (1/2/3/4)			
VNC (AP)	1/15/12/3	1/14/13/3	0.968
VNC (PVP)	2/12/9/8	2/12/9/8	0.961
VNC (EP)	0/10/10/11	0/7/13/11	0.923
Image noise (1/2/3/4)			
VNC (AP)	0/1/10/20	0/1/8/22	0.781
VNC (PVP)	0/0/9/22	0/0/7/24	0.665
VNC (EP)	0/0/11/20	0/0/8/23	0.625
Image distortion (1/2/3/4)			
VNC (AP)	0/0/0/31	0/0/0/31	NA
VNC (PVP)	0/0/0/31	0/0/1/30	NA
VNC (EP)	0/0/1/30	0/0/0/31	NA
Overall image quality (1/2/3/4)			
VNC (AP)	0/0/29/2	0/0/26/5	0.528
VNC (PVP)	0/2/26/3	0/3/25/3	0.727
VNC (EP)	0/0/23/8	0/0/20/11	0.775

Note - VNC(AP): virtual non contrast for arterial phase, VNC(PVP): virtual non contrast for portal vein phase, VNC(EP): virtual non contrast for equilibrium phase.

## 4 Discussion

This study evaluated the quantitative and qualitative characteristics of VNC images derived from triphasic dynamic liver CT (AP, PVP, and EP) using a second-generation DLCT system, by comparing them with TNC images.

The quantitative evaluation showed good agreement between the CT attenuation values of VNC images generated from all phases and those of TNC images, with the 95% LoA falling within the clinically acceptable range (±10 HU). These results suggest that second-generation DLCT-derived VNC images can reliably replace TNC images for measuring liver parenchyma, regardless of the iodine concentration in the source images. Xu et al. demonstrated a strong correlation between liver fat quantification using VNC and TNC attenuation. These results are consistent with those findings [20]. Therefore, this supports the potential omission of TNC acquisition in these clinical scenarios, leading to reduced radiation exposure. However, it should be noted that a systematic negative bias (i.e., underestimation) was observed in all VNC images. This phenomenon has been described by Laukamp et al., who observed falsely low attenuation in VNC images when contrast media was absent or low [16]. Kazimierczak et al. also reported significantly lower attenuation in delayed-phase VNC compared to TNC [21]. The magnitude of this bias was more pronounced in VNC(AP) and VNC(EP) than in VNC(PVP). Therefore, although the measured values remain within acceptable limits, clinicians should use VNC images with a clear understanding of these specific characteristics.

On the other hand, the qualitative evaluation of visual image quality revealed clear differences between the phases. These results suggest that the accuracy of iodine removal by the VNC algorithm depends on the iodine concentration present in the source image. Specifically, VNC(EP) images outperformed those VNC(AP) and VNC(PVP) in terms of vascular remnants, vessel visibility, and overall image quality. In the AP and PVP, the hepatic artery, portal vein, and hepatic parenchyma are enhanced with high iodine concentrations. Our results indicate that, even with the latest second-generation DLCT algorithms, completely separating and removing such high concentrations of iodine remains challenging. Consistent with our findings, Laukamp et al. reported that iodine subtraction in hepatic arteries was insufficient in the arterial phase using SDCT [16]. Similarly, Lehti et al. demonstrated that VNC(AP) images exhibited higher attenuation due to incomplete iodine removal compared to the venous phase [22]. Residual iodine, visualized as vascular remnants, results from this incomplete removal process. It obscures vessel contours and reduces vessel visibility [16]. Previous studies have shown that in regions with extremely high iodine concentrations, a saturation effect occurs in which the CT value reaches the upper limit of the Hounsfield scale. This reduces the difference in CT values between low and high tube voltages, leading to incomplete algorithmic material separation. Localized beam hardening and streak artifacts caused by high-concentration iodine also impair accurate iodine separation [23]. The incomplete removal of iodine resulting from these mechanisms appears as areas of high attenuation on VNC images, closely mimicking acute hemorrhage or dense calcifications. This poses a risk of diagnostic misinterpretation, such as false-positive diagnoses of hemorrhage or obscuration of true microcalcifications [24]. In contrast, substantial iodine washout from the liver parenchyma and blood vessels during the EP results in the lowest iodine concentration among the three phases. Under these low-concentration conditions, the material decomposition algorithm is less susceptible to the effects of saturation and beam hardening. This allows for more accurate iodine separation and removal. As a result, VNC(EP) images achieve the highest overall quality scores, with minimal remnant artifacts, clear vessel contours, and a visual appearance most closely resembling TNC images [15]. Previous studies using dual-energy CT (DECT) systems have widely reported that high iodine concentration is the primary cause of VNC artifacts [15], [25], [26]. This study demonstrated that iodine concentration dependency remains a limiting factor for VNC image quality even in the latest second-generation DLCT systems, as revealed by direct comparison of triphasic dynamic liver CT.

Also, no significant differences were observed among the three phases regarding image noise or image distortion. This positive finding indicates that the VNC algorithm functions stably with data from any phase, without significantly altering the fundamental physical characteristics of the image (e.g., noise texture or geometric properties). Clearly, the differences in VNC image quality are attributable primarily to the accuracy of iodine removal.

These findings have important clinical implications for the characterization of liver lesions. In clinical practice, VNC images are often used in place of TNC images to identify baseline hyper-attenuating lesions, intralesional fat, or calcifications. However, as this study demonstrated, incomplete iodine subtraction in AP and PVP images may obscure subtle lesion features or lead to misinterpretation of residual vascular enhancement as a positive finding. Therefore, VNC(EP) images, which are least affected by residual iodine, are preferable for evaluating the underlying liver parenchyma and may also provide a reliable baseline for focal liver lesion assessment.

Recently, emerging technologies such as photon-counting CT (PCCT) and AI-based VNC generation have garnered significant attention [27], [28]. While PCCT offers superior spatial and energy resolution and may mitigate VNC artifacts under high-iodine conditions, deep learning-based methods are also continuing to advance rapidly; however, the widespread availability of both technologies remains limited. In contrast, second-generation DLCT is already widely integrated into routine clinical practice. Because DLCT derives VNC images directly from standard contrast-enhanced scans without requiring additional radiation exposure or dedicated protocols, it currently represents the most practical and accessible approach for daily clinical use.

This study has several limitations. First, because this was a single-center retrospective study with a relatively small sample size (n = 31), the generalizability of our findings may be limited. We were also unable to conduct a comprehensive subgroup analysis to determine whether attenuation bias in VNC images varies with specific patient factors, such as high iodine load, body habitus, or liver disease. Therefore, future multicenter validation studies with larger cohorts are necessary to confirm these findings and strengthen their broader clinical applicability. Second, the evaluation was limited to a specific CT system and VNC algorithm; therefore, our findings may not be fully generalizable to other dual-energy CT platforms or future algorithm updates. Third, the qualitative analysis relied on subjective visual assessment. However, to minimize bias, two independent readers were employed, and inter-observer agreement was evaluated. Finally, because this study focused on evaluating hepatic parenchyma and vascular structures, we did not directly assess diagnostic performance for liver lesions, including tumor visibility and detectability. Although the superior image quality of VNC(EP) suggests clinical benefits, further research is needed to confirm the diagnostic utility of VNC(EP) for liver diseases.

In conclusion, VNC(AP), VNC(PVP), and VNC(EP) images of dynamic liver CT using a second-generation DLCT system were quantitatively comparable to TNC images, particularly for attenuation measurements such as fatty liver assessment. While these findings suggest a significant potential for omitting TNC acquisitions, clinicians and radiological technologists must fully understand the specific characteristics—both advantages and limitations—of VNC images derived from each phase and utilize them appropriately according to the diagnostic purpose.

## CRediT authorship contribution statement

**Yuji Tachibana:** Writing – original draft, Visualization, Validation, Software, Methodology, Investigation, Formal analysis, Data curation, Conceptualization. **Tomoaki Shiroo:** Resources, Investigation, Data curation. **Noritaka Kamei:** Investigation. **Yoshiki Asayama:** Writing – review & editing, Validation, Supervision, Resources, Project administration, Methodology, Investigation, Data curation, Conceptualization.

## Ethics approval

This study was approved by the Oita University Ethics Review Committee (approval number: 3211; September 1, 2025). All procedures performed in this study were in accordance with the ethical standards of the institutional research committee and the 1964 Declaration of Helsinki and subsequent amendments.

## Consent for publication

The disclosure of personal information was exempted from written consent through an internal facility review, and the information was disclosed through the website.

## Funding

This work was supported by JSPS KAKENHI Grant Number JP24K10814.

## Declaration of Competing Interest

The authors declare that they have no competing interests.

## Data Availability

The datasets used and analysed during the current study are available from the corresponding author on reasonable request.
